# *TP53*-induced glycolysis and apoptosis regulator promotes proliferation and invasiveness of nasopharyngeal carcinoma cells

**DOI:** 10.3892/ol.2014.2797

**Published:** 2014-12-12

**Authors:** ELAINE YUE LING WONG, SZE-CHUEN CESAR WONG, CHARLES MING LOK CHAN, EMILY KAI YEE LAM, LOUISA YEUNG HO, CECILIA PIK YUK LAU, THOMAS CHI CHUEN AU, AMANDA KIT CHING CHAN, CHI MAN TSANG, SAI WAH TSAO, VIVIAN WAI YAN LUI, ANTHONY TAK CHEUNG CHAN

**Affiliations:** 1 State Key Laboratory of Oncology in South China, Sir YK Pao Centre for Cancer, Department of Clinical Oncology, Hong Kong Cancer Institute and Prince of Wales Hospital, The Chinese University of Hong Kong, P.R. China; 2 Department of Health Technology and Informatics, Hong Kong Polytechnic University, Hong Kong SAR, P.R. China; 3 Department of Neuroscience, University of Minnesota, Minneapolis, MN 55455, USA; 4 Department of Pathology, Queen Elizabeth Hospital, University of Hong Kong, Hong Kong SAR, P.R. China; 5 Department of Anatomy, University of Hong Kong, Hong Kong SAR, P.R. China; 6 Department of Pharmacology and Pharmacy, University of Hong Kong, Hong Kong SAR, P.R. China

**Keywords:** nasopharyngeal carcinoma, *TP53*-induced glycolysis and apoptosis regulator, cell growth, invasiveness, mesenchymal

## Abstract

The *TP53*-induced glycolysis and apoptosis regulator (TIGAR) is the protein product of the p53 target gene, *C12orf5*. TIGAR blocks glycolysis and promotes cellular metabolism via the pentose phosphate pathway; it promotes the production of cellular nicotinamide adenine dinucleotide phosphate (NADPH), which leads to enhanced scavenging of intracellular reactive oxygen species, and inhibition of oxidative stress-induced apoptosis in normal cells. Our previous study identified a novel nucleoside analog that inhibited cellular growth and induced apoptosis in nasopharyngeal carcinoma (NPC) cell lines via downregulation of TIGAR expression. Furthermore, the growth inhibitory effects of c-Met tyrosine kinase inhibitors were ameliorated by the overexpression of TIGAR in the NPC cell lines. These results indicate a significant role for TIGAR expression in the survival of NPCs. The present study aimed to further define the function of TIGAR expression in NPC cells. In total, 36 formalin-fixed, paraffin-embedded NPC tissue samples were obtained for the immunohistochemical determination of TIGAR expression. The effects of TIGAR expression on cell proliferation, NADPH production and cellular invasiveness were also assessed in NPC cell lines. Overall, TIGAR was overexpressed in 27/36 (75%) of the NPC tissues compared with the adjacent non-cancer epithelial cells. Similarly, TIGAR overexpression was also observed in a panel of six NPC cell lines compared with normal NP460 hTert and Het1A cell lines. TIGAR overexpression led to increased cellular growth, NADPH production and invasiveness of the NPC cell lines, whereas a knockdown of TIGAR expression resulted in significant inhibition of cellular growth and invasiveness. The expression of the two mesenchymal markers, fibronectin and vimentin, was increased by TIGAR overexpression, but reduced following TIGAR-knockdown. The present study revealed that TIGAR overexpression led to increased cellular growth, NADPH production and invasiveness, and the maintenance of a mesenchymal phenotype, in NPC tissues.

## Introduction

The *TP53*-induced glycolysis and apoptosis regulator (TIGAR), which contains six coding exons and two p53 binding sites, is the protein product of a p53 target gene, *C12orf5*, located on chromosome 12p13-3 (1). Although p53 has already been established as a tumor suppressor protein, recent studies have demonstrated that by promoting cellular metabolism and blocking glycolysis via the TIGAR-mediated pentose phosphate pathway (PPP), p53 is also able to control cellular metabolism. In normal cells, this results in increased nicotinamide adenine dinucleotide phosphate (NADPH) production, enhanced scavenging of intracellular reactive oxygen species (ROS) and inhibition of oxidative stress-induced apoptosis. Therefore, the activation of TIGAR by p53 promotes an antioxidant response that enables cells to survive during stressful conditions (2–4).

However, recent studies have revealed that deregulated TIGAR expression enhances the development of cancer by promoting the survival of cancer cells. In breast cancer, TIGAR expression was identified to protect the cells from undergoing apoptosis (5). In multiple myeloma cells, TIGAR was revealed to be necessary for the maintenance of redox homeostasis, whereas the downregulation of TIGAR resulted in myeloma cell death (6). In cases of hepatocellular carcinoma, the suppression of TIGAR expression was identified to induce apoptosis and autophagy (7). Furthermore, in a mouse model of intestinal adenoma, TIGAR-deficient mice exhibited reduced adenoma size and tumor burden compared with wild-type mice. Overall, no significant difference was observed in the number of tumors, which suggested that TIGAR is primarily involved in tumor progression, rather than tumor initiation (4). In addition, the reduced tumor burden was correlated with an improved survival rate of the TIGAR-deficient mice (4). This evidence suggested that TIGAR confers a protective function to cancer cells within multiple tissue types.

Nasopharyngeal carcinoma (NPC) is a metastatic and highly invasive Epstein-Barr virus (EBV)-associated cancer of the nasopharynx. The disease is particularly prevalent in China, with an annual incidence of up to 25 cases per 100,000 individuals (8). At diagnosis, >60% of patients present with advanced stages of the disease, which due to distant recurrence or metastasis, are commonly unresponsive to treatment (9). Therefore, additional effective therapies are required for the treatment of NPC.

Our previous study revealed that a novel nucleoside analog inhibited cellular growth and induced apoptosis in NPC cell lines via downregulation of TIGAR expression (10). A further study demonstrated that the growth inhibitory effects of c-Met tyrosine kinase inhibitors were ameliorated by the overexpression of TIGAR in NPC cell lines (11). These results indicate a significant role for TIGAR expression in the survival of NPC cells. However, functional studies examining the role of TIGAR in NPC are lacking. The present study sought to investigate the expression pattern of TIGAR in NPC tumor tissues, and to analyze the consequences of TIGAR overexpression and knockdown on NPC cell growth and invasion.

## Materials and methods

### Antibodies

The antibodies used in the present study were rabbit anti-human TIGAR polyclonal antibody (cat no. ab37910, dilution, 1:8,000; Abcam, Cambridge, UK), rabbit anti-human fibronectin polyclonal antibody (cat no. sc-9068, dilution, 1:2,000; Santa Cruz Biotechnology Inc., Dallas, TX, USA), mouse anti-pig vimentin monoclonal antibody (cat no. V6389, dilution, 1:1,000; Sigma-Aldrich, St. Louis, MO, USA), mouse anti-chicken actin monoclonal antibody (cat no. MAB1501, dilution, 1:100,000; Merck Millipore, Darmstadt, Germany), goat anti-mouse IgG polyclonal antibody HRP conjugate (cat no. 170-6516, dilution, 1:10,000; Bio-Rad Laboratories, Hercules, CA, USA) and goat anti-rabbit IgG polyclonal antibody HRP conjugate (cat no. 81-6120, dilution, 1:10,000; Thermo Fisher Scientific, Waltham, MA, USA).

### Immunohistochemistry (IHC) staining

In total, 36 formalin-fixed, paraffin-embedded specimens of undifferentiated NPC, with adjacent normal epithelium, were retrieved from the archives of the Department of Pathology, Queen Elizabeth Hospital (Hong Kong, China).

The 4-μm thick, formalin-fixed, paraffin-embedded serial tissue sections were cut, and antigen retrieval was performed at 100°C for 25 min using Bond Epitope Retrieval Solution 2 on the Bond-max automated immunostainer (Leica Microsystems, Wetzlar, Germany). The immunostaining was performed using a polymer detection system in the immunostainer with a rabbit polyclonal TIGAR antibody (1:500 dilution; Abcam), according to the manufacturer’s instructions. Lymphoid cells were used as internal positive controls for TIGAR expression, and negative controls were constructed by replacing the antibody with Tris-buffered saline. The stained slides were analyzed in five fields using a light microscope (Leica DMLS; Leica Microsystems) at ×400 magnification. The two independent observers were without knowledge of the clinical outcomes, and in the case of a disagreement, a consensus was reached following thorough discussion and slide examination using a multi-headed microscope (Leica DMLS; Leica Microsystems). In total, ~250 cells were counted in each field, and therefore at least 1,250 cells were counted for each tissue specimen. All the slides were scored semi-quantitatively and expressed as an IHC score by multiplying the percentage of positive cells by the staining intensity, as previously described (12). The staining intensity was scored as follows: 0, negative; 1, weak; 2, moderate; 3, strong; and 4, very strong. The IHC score ranged from 0 to 400.

### Cell lines

The HONE-1 NPC cell line was derived from patients with poorly-differentiated NPC (13,14). The HONE-1-EBV cell line was derived from the introduction of the EBV genome into the HONE-1 parental NPC cell line (15). The prototype latent membrane protein 1 (LMP1) was cloned from a B95.8 cell line. All the NPC cell lines were maintained in RPMI-1640 medium supplemented with 10% fetal bovine serum (Thermo Fisher Scientific), 100 U/ml penicillin, 100 μg/ml streptomycin and 1 mm sodium pyruvate (Thermo Fisher Scientific). The cells were cultured at 37°C with 5% CO_2_ in a cell culture incubator. The HONE-1-EBV cell line was maintained in a selection media containing 400 μg/ml G418 reagent (Thermo Fisher Scientific). The NP460 hTert cell line (obtained from Professor S.W. Tsao, Department of Anatomy, University of Hong Kong, Hong Kong, China) was maintained in a 1:1 dilution of Epilife medium and defined keratinocyte serum-free medium (KSFM; Thermo Fisher Scientific). The Het-1A cells were obtained from the American Type Culture Collection (Manassas, VA, USA), and maintained in the KSFM supplemented with 25 μg/ml bovine pituitary extract and 0.15 ng/ml epidermal growth factor (Thermo Fisher Scientific). The HONE-LMP1 TIGAR-expressing cell line was established by the transient co-transfection of the TIGAR-overexpressing plasmid, or the respective control (OriGene, Rockville, MD, USA), into the parental HONE-1-LMP1 cell line using a pcDNA3.1(+) vector (Thermo Fisher Scientific). The cell line was then subjected to G418 selection for 3 months for single clone development, and maintained in a selection media containing 400 μg/ml G418 (Thermo Fisher Scientific).

### Western blotting

The cell lysates were prepared as previously described (16). For the preparation of the NPC tumor biopsy lysates, six frozen endoscopy-guided biopsies, obtained from treatment-naïve NPC patients, were collected at the time of diagnosis. The patients consented to tissue collection for research purposes at the Tumor Bank, Department of Clinical Oncology, The Chinese University of Hong Kong (Hong Kong, China) according to the approved Ethics Approval of Research Protocol. The frozen tumor samples were homogenized using a pellet-pestle, disposable, cordless, hand-held homogenizer (Sigma-Aldrich) on ice in a western lysis buffer containing 1.25 mm DTT, 5 mm phenylmethanesulfonylfluoride, 30 μg/ml leupeptin and 30 μg/ml aprotinin. In total, 50 μg protein was subjected to SDS-PAGE and immunoblotting, as previously described (17). Actin was used as the loading control.

### Plasmid, siRNA and transfection

The pCMV-XL5 plasmid was used as the control vector for stable clone development in the transfection experiments. The HONE-1-LMP1 cells were plated at a density of 0.8×10^5^ cells per well in a 10-cm^2^ plate. After 24 h, the cells were co-transfected using the pcDNA3.1(+) vector with 10 μg pCMV-XL5- or TIGAR-overexpressing plasmids (OriGene). Subsequent to 48 h of transfection, the cells were subjected to 400 μg/ml G418 selection for 3 months for clone development. The HONE-1-LMP1 cells were plated at a density of 1.2×10^5^ cells per well in a 6-well plate. After 24 h, the cells were transfected with 20 nM TIGAR or negative control siRNA (GE Healthcare Dharmacon, Inc., Lafayette, CO, USA), using Lipofectamine 2000 (Thermo Fisher Scientific). Subsequent to 48 h of transfection, the cells were harvested for western blotting to confirm TIGAR-knockdown, and for the cell counting and Matrigel invasion assays.

### Cell viability assay

The viable cell number of the stable clone- and siRNA-transfected cells was determined by a trypan blue exclusion assay. The cells were harvested and the cell number was determined by counting with 50% trypan blue (Thermo Fisher Scientific) on a hemocytometer. The experiments were performed at least three times, and triplicate wells were counted in each experiment.

### Intracellular NADPH determination

The cellular NADPH production was determined using the EnzyChrom^TM^ NADP^+^/NADPH assay kit (Bioassay Systems, Hayward, CA, USA), as previously described (10). The protein concentration of the samples was determined using protein quantification, as previously described (17). The NADPH concentration was normalized to the total protein, and presented as μM/min/mg total protein. In total, at least three independent experiments were performed.

### Matrigel invasion assay

Matrigel-coated Boyden inserts, with a pore size of 8 μm, were used for the invasion assay (BD Biosciences). The cells were seeded into the upper chamber at a density of 7×10^4^ cells and maintained in serum-free medium. The cell-containing chamber was immersed in a lower chamber containing complete medium. The cells were incubated for 24 h at 37°C in a 5% CO_2_ incubator. The non-invaded cells, which remained in the upper chamber, were removed with a cotton swab. The invaded cells were then stained with 1% toluidine blue O in 1% borax (Sigma-Aldrich) and counted under a microscope (magnification, ×200). In total, 10 random fields were counted, and each experiment was performed in triplicate.

### Statistical analysis

Statistical analyses were performed using PRISM4 software (GraphPad, San Diego, CA, USA). P-values were obtained using an unpaired t-test with Welch’s correction. P<0.05 was considered to indicate a statistically significant difference.

## Results

### TIGAR is overexpressed in NPC tumors and cell lines

**Figure 1:**
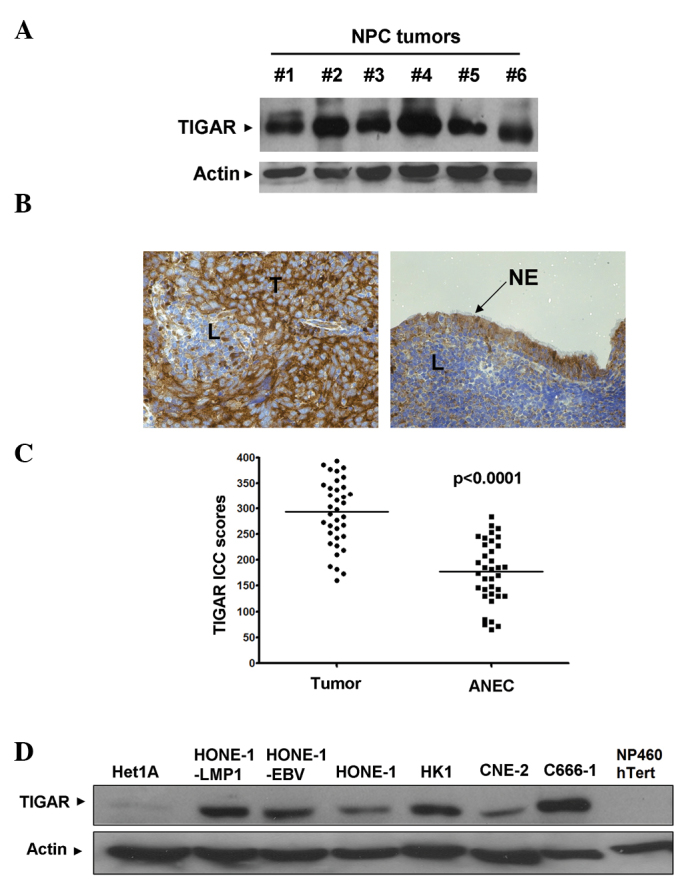
*TP53*-induced glycolysis and apoptosis regulator (TIGAR) is overexpressed in nasopharyngeal carcinoma (NPC) tumors and cell lines. (A) Western blot analysis revealing the expression of TIGAR in six NPC tumor biopsies. (B) Immunohistochemistry (IHC) staining revealing TIGAR overexpression in NPC (left) and adjacent normal epithelial cells (ANECs; right) (magnification, ×400). (C) IHC scores of TIGAR expression in tumor cells and ANECs, with a black horizontal line revealing the median score for each group. (D) TIGAR expression in multiple NPC cell lines with various differentiation statuses compared with the normal NP460 hTert nasopharyngeal epithelial and Het1A esophageal epithelial cell lines. HONE-1, HONE-1-LMP1, HONE-1-EBV and CNE-2 were poorly-differentiated, HK-1 was well-differentiated and C666-1 was undifferentiated. Actin served as the loading control. T, tumorous region; L, lymphocytes; NE, normal epithelium.

TIGAR protein expression was detected by western blotting in all the NPC tissue samples (Fig. 1A). In order to gain an understanding of the expression pattern of TIGAR in NPC tumors, IHC was used. Of the 36 NPC specimens, 27 (75%) demonstrated higher TIGAR IHC scores compared with the respective adjacent normal epithelial cells (ANECs) (Fig. 1B and C). With respect to the remaining nine NPC specimens, five (13.9%) demonstrated similar TIGAR IHC scores (±5) and 4 (11.1%) exhibited lower TIGAR IHC scores compared with the respective ANECs. In summary, the median TIGAR IHC scores of the tumor cells and the ANECs were 293.5 and 178, respectively. Furthermore, the differences between the TIGAR IHC scores of the tumor and adjacent normal cells were statistically significant (P<0.0001; Mann-Whitney U test).

Similar to the results obtained from the IHC, western blotting identified TIGAR overexpression across a panel of six NPC cell lines of varying differentiation statuses (HK-1 was from differentiated from NPC; HONE-1, HONE-1-LMP1, HONE-1-EBV and CNE2 were from poorly-differentiated NPC; and C666-1 was from undifferentiated NPC) compared with the normal NP460 hTert nasopharyngeal cell line and the normal Het-1A esophageal epithelial cell line (Fig. 1D). TIGAR expression appeared to be increased by EBV infection or expression of the EBV LMP1 oncoprotein, as HONE-1-EBV and HONE-1-LMP1 expressed higher levels of TIGAR compared with the parental HONE-1 cells.

### TIGAR promotes cell proliferation in NPC cells

**Figure 2:**
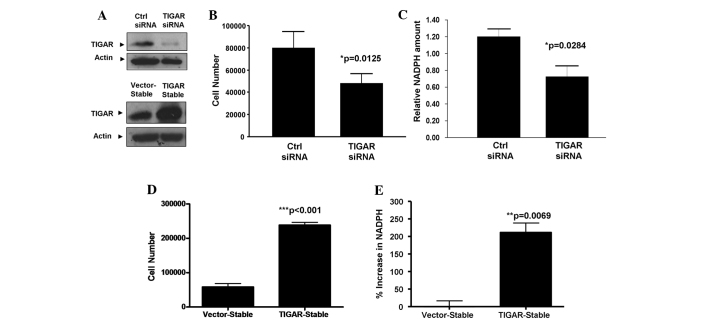
*TP53*-induced glycolysis and apoptosis regulator (TIGAR) regulates nasopharyngeal carcinoma (NPC) cell growth and nicotinamide adenine dinucleotide phosphate (NADPH) production. (A) Western blot analysis revealing the knockdown (upper figure) and upregulation (lower figure) of TIGAR expression following transfection with TIGAR small interfering RNA and TIGAR-overexpressing plasmids, respectively. Knockdown of TIGAR (B) inhibited NPC cell growth and (C) depleted cellular NADPH production. The stable expression of TIGAR (D) promoted cellular proliferation and (E) increased NADPH production. Results are expressed as the mean ± standard error of the mean. ^*^P<0.05, ^**^P<0.01 and ^***^P<0.001 vs. the respective control group.

The biological consequences of TIGAR upregulation in NPCs are unclear. Therefore, to investigate the functional role of TIGAR within NPC cells, endogenous TIGAR expression was knocked down in HONE-1-LMP1 NPC cells (Fig. 2A, upper panel). The results revealed that the knockdown of TIGAR expression led to significant growth inhibition of the HONE-1-LMP1 cell line (P=0.0125; unpaired t-test; Fig. 2B). To further confirm the regulatory effect of TIGAR upon NPC cell proliferation, stable cells that overexpressed TIGAR were created with the HONE-1-LMP1 background (Fig. 2A, lower panel). As demonstrated in Fig. 2C, HONE-1-LMP1-TIGAR cells exhibited a four-fold increase in cell growth compared with the HONE-1-LMP1-vector control cells (P<0.001; unpaired t-test). These findings indicate that TIGAR is capable of regulating NPC cell proliferation.

### TIGAR expression promotes NADPH production in NPC cells

TIGAR has been reported to increase the production of cellular NADPH, an antioxidant that is required for ROS scavenging and the inhibition of apoptosis through the PPP (2). In order to investigate whether TIGAR regulates NADPH production within NPC cells, TIGAR expression was knocked down in HONE-1-LMP1 cells by siRNA transfection. The results in Fig. 2D reveal that the silencing of TIGAR in the HONE-1-LMP1 cells led to reduced amounts of cellular NADPH (P=0.0284; unpaired t-test). Furthermore, the overexpression of TIGAR in the HONE-1-LMP1-TIGAR stable cells led to a two-fold increase in NADPH production compared with the vector-stable cells (P=0.0069; unpaired t-test; Fig. 2E). These results indicate that TIGAR expression is capable of promoting NADPH production in NPC cells.

### TIGAR promotes the invasiveness of NPC cells

**Figure 3:**
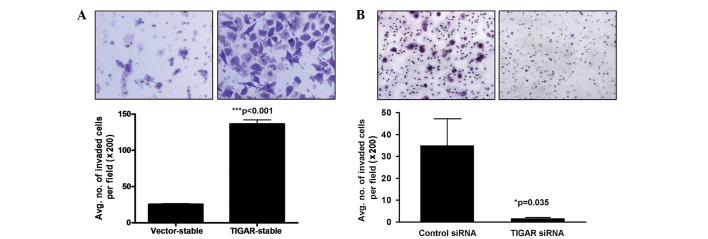
*TP53-*induced glycolysis and apoptosis regulator (TIGAR) expression promotes cellular invasiveness. (A) A greater number of stable TIGAR-expressing nasopharyngeal carcinoma cells, compared with vector-transfected cells, invaded through the Matrigel layer. (B) A reduced number of TIGAR siRNA-transfected cells, compared with control siRNA-transfected cells, invaded through the Matrigel layer. Cells invading through the Matrigel inserts were fixed, stained and counted under a microscope (magnification, ×200). Results are expressed as the mean ± standard error of the mean. ^***^P<0.001 and ^*^P=0.035 vs. the respective control group.

In order to investigate whether TIGAR promotes the malignant properties of NPC cells, the effects of TIGAR overexpression on the invasiveness of the HONE-1-LMP1-TIGAR NPC cell line, compared with the vector stable cells, was examined. As revealed in Fig. 3A, TIGAR overexpression led to a five-fold increase in the number of NPC cells that invaded through the Matrigel (P<0.001; unpaired t-test). Furthermore, knockdown of TIGAR expression in HONE-1-LMP1 cells by RNAi reduced the number of cells that invaded through the Matrigel by ten-fold (P=0.035; unpaired t-test; Fig. 3B). These findings demonstrate that TIGAR is involved in the promotion of NPC cell invasion.

### TIGAR induces the expression of mesenchymal markers in NPC cells

**Figure 4:**
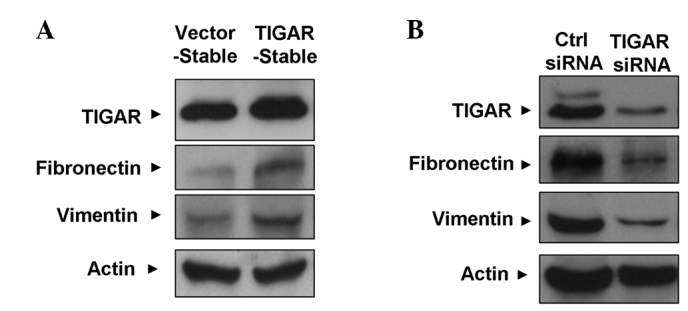
*TP53*-induced glycolysis and apoptosis regulator (TIGAR) induces the expression of mesenchymal markers. (A) TIGAR overexpression induced an increase in the expression levels of the mesenchymal markers fibronectin and vimentin, whereas (B) TIGAR-knockdown suppressed the expression of fibronectin and vimentin. Actin was used as the loading control.

During invasion and metastasis, tumor cells often undergo a process known as epithelial-mesenchymal transition (EMT) (18). In order to investigate whether TIGAR-induced NPC cells also undergo this process, the expression of several epithelial and mesenchymal markers was examined by western blotting. Fig. 4A reveals that the expression of the mesenchymal markers, fibronectin and vimentin, was upregulated in the HONE-1-LMP1-TIGAR cells. This finding is consistent with the changes associated with a mesenchymal phenotype. Conversely, Fig. 4B demonstrates that the expression of fibronectin and vimentin was reduced upon TIGAR-knockdown. Together, these results indicate that TIGAR promotes the expression of mesenchymal markers in NPC cells, which may explain the increased invasiveness of TIGAR-expressing NPC cells.

## Discussion

The present study demonstrated that TIGAR expression is upregulated in NPC tissues and cell lines. To the best of our knowledge, this study is the first to examine the expression of TIGAR in NPC tissues. The significant increase in TIGAR expression in the tumor cells compared with ANECs may indicate that TIGAR is involved in the development of NPC. The results correspond with those from recent studies in which TIGAR was revealed to be involved in the tumorigenesis of intestinal cancer and glioblastomas (4,19). This evidence suggests that TIGAR is a potential oncogene involved in various cancers. Further studies, which will include a larger cohort of specimens, are required to validate these findings and investigate the association between TIGAR expression and the clinical and histopathological features of NPC. Furthermore, functional tests, performed in a number of cell lines, are required in order to examine whether TIGAR is a potential therapeutic target for NPC.

In the HONE-1-LMP1 EBV-related NPC cell line, the overexpression of TIGAR promoted cellular NADPH production, proliferation and invasion, and resulted in a concomitant upregulation of fibronectin and vimentin expression, which was indicative of a mesenchymal phenotype. Conversely, the knockdown of TIGAR by siRNA led to a reduction in cellular proliferation, invasiveness and NADPH production, and the reduced expression of fibronectin and vimentin. Together, these findings indicate that TIGAR promotes NPC cellular survival and invasiveness, and induces a mesenchymal phenotype.

TIGAR is the protein product of a p53 target gene, it exhibits fructose-2 and 6-bisphosphatase activity, and lowers the cellular levels of fructose-2,6-bisphosphate. This inhibits glycolysis and promotes the PPP, which increases the production of NADPH, and reduces the expression of glutathione (2). The synthesis of biomolecules and protection against oxidative stressors are processes necessary for cellular survival and that rely upon the antioxidant, NADPH (20,21). The results from the present study revealed that the overexpression of TIGAR upregulates cellular NADPH production. One of the major sources of oxidative stress within cells originates from the accumulation of ROS, which have the potential to lead to cell cycle arrest or cell death. In order to counteract the detrimental effects of ROS, cells increase the production of antioxidants, such as NADPH, which is a major source of the cellular reducing capability. In line with the pro-survival role of NADPH, the present study identified that TIGAR overexpression increased NPC cell proliferation, while TIGAR-knockdown inhibited NPC cell proliferation. This demonstrated the specificity of the TIGAR-mediating growth effect in NPC cells. These results are consistent with those from a previous study, which identified that TIGAR prevented cell death via modulation of PPP activity (2). In the study, TIGAR reduced ROS levels and protected the cells from ROS-associated apoptosis, while TIGAR-knockdown sensitized the cells to p53-induced cell death. Consistent with the role of TIGAR in NPC cell survival, our previous studies revealed that TIGAR expression reversed the anti-proliferative effects of c-met tyrosine kinase inhibition on NPC cell growth (10,11). In summary, these findings provide significant evidence to support the role of TIGAR in NPC cell survival.

In the present study, TIGAR overexpression promoted NPC cellular invasion through the Matrigel, an effect that was ameliorated by the siRNA-mediated knockdown of TIGAR. This novel finding is significant in that distant metastases are the predominant cause of treatment failure in patients with NPC (8). The present study revealed that a downregulation of TIGAR expression inhibited NPC cell invasiveness. This finding may provide a novel therapeutic target for the treatment of patients with advanced stages of NPC, and one that may improve clinical prognoses. Furthermore, the regulation of TIGAR to prevent NPC cell invasion into surrounding tissues and prevent progression of the disease to more advanced stages may be investigated in future studies. At present, the precise mechanisms that underlie TIGAR-mediated NPC cell invasion are unclear. During tumor progression, carcinoma cells lose epithelial properties and undergo EMT to gain invasiveness. This process promotes tumor intravasation into lymph or blood vessels, and metastasis in distant organs (18). In cases of NPC, invasiveness and metastasis have been associated with EMT (22,23). In order to gain insight into the molecular changes associated with TIGAR-induced invasion, alterations in the expression of EMT markers were investigated within the present study. The results demonstrated that the overexpression of TIGAR in the HONE-1-LMP1 NPC cell line upregulated the expression of the mesenchymal markers, fibronectin and vimentin, whereas the silencing of TIGAR led to downregulation. The role of TIGAR in the regulation of EMT is supported by a recent study, which revealed that the modulation of the p53/TIGAR pathway induced changes in the EMT status of a cervical carcinoma cell line (24). These findings suggested that the expression of TIGAR promoted NPC cellular invasion by initiating changes in the EMT phenotype.

In conclusion, the present study identified that TIGAR is overexpressed in NPC, where the protein is involved in the promotion of cellular proliferation, NADPH production and invasion, and in the expression of mesenchymal markers. Given that the involvement of TIGAR within these cellular processes may promote tumor progression, further investigations that examine how TIGAR supports NPC tumor growth, and the associated molecular pathways, are warranted.
